# Hairy Roots Cultures from the Slow-Growing and Protected *Panax ginseng*, *Leontopodium alpinum* and *Gynostemma pentaphyllum* as an Alternative Source of Antimicrobials

**DOI:** 10.3390/antibiotics15090905

**Published:** 2026-09-14

**Authors:** Olivier Braissant, Virginie Flury, Muriel Jobin, Sophie Hu

**Affiliations:** 1 Department Biomedical Engineering, University of Basel, Hegenheimermattweg 167 B/C, CH-4123 Allschwil, Switzerland; 2 DXC RT AG, Gewerbestrasse 7a, CH-4147 Aesch, Switzerland

**Keywords:** *Panax ginseng*, *Leontopodium alpinum*, isothermal microcalorimetry, hairy root culture

## Abstract

**Background/Objectives**: Although the potential of hairy roots in secondary metabolite including antimicrobal production has been demonstrated in many laboratories, the scale-up to industrial production might lead to undesired phenomena affecting the final products. **Methods**: Here, the hairy root cultures of a pilot plant was investigated for antimicrobial properties. Methanolic extract of *Panax ginseng* Mey., *Leontopodium alpinum* Cass., and *Gynostemma pentaphyllum* Thunb. that are slow-growing, hard-to-cultivate and endangered species, were tested for antimicrobial activity using isothermal microcalorimetry and the Kirby–Bauer assay. **Results**: Antimicrobial effects were detected for *P. ginseng* and *L. alpinum* extract with a minimum inhibitory concentration (MIC) at 3.6 mg·mL^−1^ and 15 mg·mL^−1^ respectively. *G. pentaphyllum* showed only minimal inhibition at high concentration and appeared to act as a prebiotic at sub-inhibitory concentrations instead. The Kirby–Bauer assays supported these results. **Conclusions**: Industrial callus culture and hairy root production are a valuable alternative to the use of field-grown plants for the production of antimicrobials and other metabolites. Effects similar to those of their field-harvested counterpart described in many pharmacopeias were observed. Finally, isothermal microcalorimetry provides a valuable tool as it can rapidly, quantitatively and accurately monitor antimicrobial efficacy even in dark or cloudy samples.

## 1. Introduction

Hairy root technology derives from the attention that was paid to the so called “hairy root disease” which was responsible for substantial losses in fruit production in the early 20th century [1]. Nowadays, what was once a disease due to *Rhizobium* (formerly *Agrobacterium*) is now recognized as a valuable natural process to produce plant secondary metabolites for different industries belonging to the pharmaceutical, cosmetics, or even food sectors [2]. Indeed, plants are amazing reservoirs of secondary metabolites including alkaloids, terpenoids, steroids, quinones, and phenylpropanoids among others [3] and using hairy root technology combined with large scale bioreactor production has shown to be able to deliver compounds with anti-arthritis, antioxidant, anti-inflammatory, antimicrobial and antitumorous activities [4]. Many of these products are difficult to produce as organic synthesis is too expensive or the biosynthetic pathways are often so complex that transferring them to a microorganism is not trivial or feasible [5], making hairy root technology a valuable and commercially viable alternative. Furthermore, many natural products are known to come from slow-growing, hard-to-cultivate, or endangered plant species [5]. Indeed, approximately 25% of the drugs worldwide are derived from plants, with 121 of such active compounds currently in use. Of the 252 essential drugs listed by the world health organization (WHO), 11% are only of plant origin and many are derived from vegetal precursors (e.g., digoxin, quinine, vincristrine, atropine, morphine). The high demand for these products has led to shortages, and even the exhaustion of some medicinal plant resources. In addition, to make the situation even worse, pollution is a growing concern in many places and the biomass extracted might require further processing to remove the soil-accumulated pollutants [6,7]. In this context, the ability to produce cleaner biomass in more reproducible conditions might also influence the quality of the final product or at least ease its purification.

However, there is a difference between lab scale and industrial scale and the scale-up from the first one to the latter might lead to undesired phenomena affecting the production of the secondary metabolites. In this study, we investigate the production of a pilot plant able to produce up to tons of hairy roots per year with respect to their antimicrobial properties. We focused on three species from the Swiss, United States of America (US) and Chinese pharmacopeia with important commercial interest and/or because of their protected status (*Panax ginseng* Mey., *Leontopodium alpinum* Cass., and *Gynostemma pentaphyllum* Thunb.). We hypothesize that the hairy roots conserve a good production of antimicrobial and that antimicrobial properties of larges batches produced in an industrial manner are similar to the literature values found for field-grown counterparts. In this study, antimicrobial activity was monitored using isothermal microcalorimetry that provides real-time measures of metabolism and growth of microbes even in dark or cloudy media and thus allows us to monitor growth delays and/or the decreased growth rate accurately [8,9].

## 2. Results

**Figure 1:**
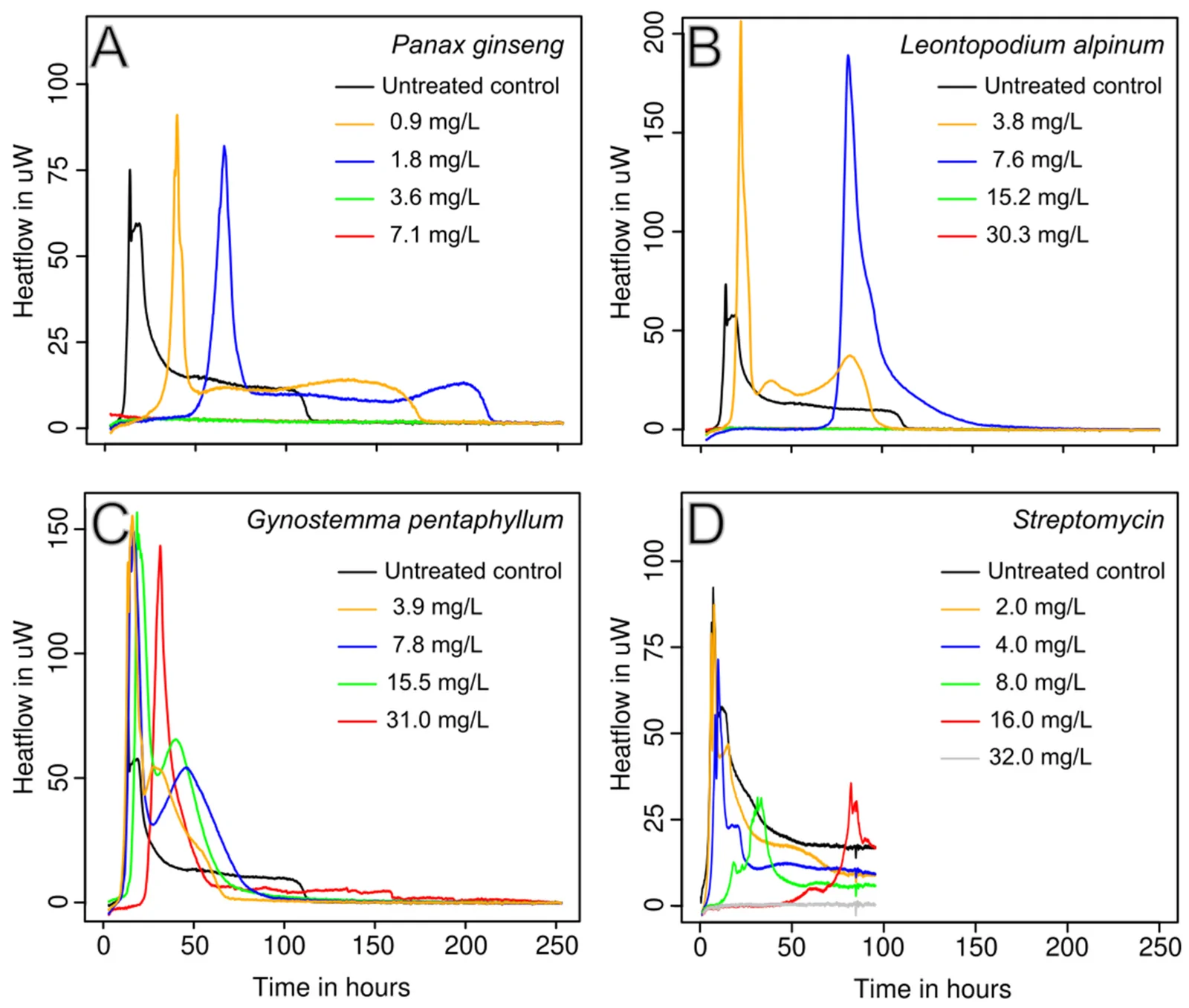
Heatflow pattern representing the metabolic activity of *S. epidermidis* with increasing concentration of hairy root extracts. *P. ginseng* (**A**); *L. alpinum* (**B**); *G. pentaphyllum* (**C**); and streptomycin (as reference antimicrobial—(**D**)). Note the strong delay in growth for *P. ginseng* as well as the strong increase in metabolic activity for *G. pentaphyllum* without growth delay.

In the calorimeters, the 50% ethanol added for the dissolution of the dried extract did not significantly alter the growth of *S. epidermidis* independently of the concentration used (i.e., maximum final concentration = 2.5%). As a consequence all controls were pooled for further comparisons. The calculated growth parameters showed that two out of the three hairy root extracts showed good antimicrobial properties (Figure 1A–C), when compared to streptomycin (Figure 1D) (Supplementary Table S1). The MICs were determined as the first concentration that fully inhibited heat production resulting in a flat signal at baseline (i.e., close to 0 µW) for the duration of the measurements. *P. ginseng* and *L. alpinum* extract showed MICs at 3.6 mg·mL^−1^ and 15 mg·mL^−1^, respectively (Figure 1A). The *P. ginseng* hairy root extract’s inhibitory effect was visible mostly through the duration of the lag phase which increased from 2 h for the control up to 40 h for the treated samples. The growth rate remained constant at sub-inhibitory concentrations of extract.

Similarly, the *L. alpinum* hairy root extract showed a similar pattern with an overall increase in the lag phase and a growth rate remaining rather constant at sub-inhibitory concentrations of extract (Figure 1B). At the lowest concentration a minor increase in growth rate and heat production was observed with a slight increase in the lag phase (from 2 to 6 h lag). Again, the Edelweiss (*L. alpinum*) extract is known to contain many antimicrobial compounds such as leontopodic acid, linoleic acid, linolic acid, but also sesquiterpenes (isocomene, b-isocomene, and silphinene) and coumarins (5-hydroxyobliquin) that have been identified as potent antimicrobials.

Overall these results are in line with previous observations on extract from harvested plants obtained by conventional culture which are both known for their antimicrobial effect. The Kirby–Bauer disk diffusion assay confirmed the results from the isothermal microcalorimetry. A clear inhibition zone was visible for both extracts. The diameter of the inhibition zone was 21 ± 1 mm (n = 4) and 21 ± 3 mm (n = 4) for *P. ginseng* and *L. alpinum*, respectively. Those 2 extracts compared well with streptomycin showing an inhibition zone of 17 ± 1 mm (n = 5) for 10 μg disks (Table 1).

The last hairy root extract from *G. pentaphyllum* did not show any antimicrobial activity at low concentrations. On the contrary it showed some growth-promoting effect with an increased growth rate and heat production (Figure 1C). Still, at the highest concentration, an increase in the lag phase from 2 to 17 h was observed; however, growth still occurred within 24 h, thus not reaching MIC. The Kirby–Bauer disk diffusion assay confirmed the antimicrobial effect at the highest concentration only, with the smallest inhibition zone of 13 ± 1 mm (n = 4) (note that no inhibition zone was visible for lower concentrations) (Table 1).

## 3. Discussion

Our results demonstrated that the hairy roots extracts could be valuable alternatives to field-grown plants. For example it takes up to 4–5 years to get a ginseng root in the field as several kilos can be produced in a month using hairy roots technology. More importantly, our results show consistency with the MIC of various extracts of these plants tested in previous studies. Unfortunately the literature is limited and often lacks necessary details. Still, in the present study, the extract of *P. ginseng* showed antimicrobial efficacy with an MIC of 3.6 mg·mL^−1^. This is in line with previous results where ethanol extract had an MIC of 90 mg·mL^−1^ against *S. aureus* [10]. Similarly aqueous extracts of ginseng lead to an MIC of 1.25–2.5 mg·mL^−1^ against various strains of *P. aeruginosa* [11]. Refined ginsenosides show a lower MIC around 0.1 mg/mL against various Gram-positive and Gram-negative bacteria [12]. Considering the variation in MIC through different species and strains, our results for *P. ginseng* methanolic extracts fit well with the literature data. Still, care should be taken when comparing those results as different extraction methods, bacterial strains, and antimicrobial assays have been used.

Similarly, the extract of *L. alpinum* showed antimicrobial efficacy with an MIC at 15 mg/mL. This is in line with early results showing that dichloromethane crude extract had antimicrobial properties [13,14] although no MIC was monitored for those crude extracts. Still the study showed that linolic and linolenic acids had the most important antimicrobial effect, with an MIC of up to 4 mg·mL^−1^, showing activity even against the multiresistant *S. aureus* strain DSM 13661. In later studies, the extracts obtained using a solvent for extraction (alcohol, petroleum-ether, ethylacetate or their combinations) also showed inhibitory effects against *Staphylococcus aureus* with an MIC of 0.14 mg·mL^−1^ L [15]. The essential oil obtained through distillation using a Clevenger-type apparatus showed good inhibition area in the Kirby–Bauer test at 10 mg·mL^−1^ [16]. Also not related to hairy roots, extracts from *L. alpinum* callus cell cultures containing phenolic fraction, 55% of which were leontopodic acids [17], also showed antimicrobial efficacy, thus emphasizing that cell cultures and hairy roots culture are valuable tools in the production of plant-based antimicrobial compounds. Again, care should be taken when comparing those results as different extraction methods, bacterial strains, and antimicrobial assays have been used. Furthermore some data for comparison come from closely related *Leontopodium leontopodioides* and *Leontopodium longifolium* extracts. So further work with hairy roots from *L. alpinum* might be necessary.

With respect to the antimicrobial compounds present in the hairy root extracts, in vitro studies have confirmed that for *P. ginseng*, ginsenosides belonging to the saponin family are mainly responsible for such an effect [18,19]. In our context, previous analyses of the hairy root biomass showed the presence of ginsenosides. The following Re, Rg, Rf, Rc, Rd, Rb1 and Rb2 ginsenosides were detected and their concentration was at least as high as what could be found in field-grown plants (see Table 2—[20,21,22]).

Similarly for *L. alpinum*, recent laboratory studies have also demonstrated that many antimicrobial compounds such as linolic, linolenic acids and leontopodic acid could be identified in both the harvested plant tissues and hairy root extracts [13,23]. As for *P. ginseng*, it seems that some of the valuable antimicrobial compounds of *L. alpinum* are produced in higher amounts in cell cultures than in field-grown plants [17]. Also, our observation seems to show that some of these compounds could be released in the used growth medium and further extracted from it (personal observations).

The last hairy root culture investigated was *G. pentaphyllum* and it did not show antimicrobial efficacy (or at concentration so high that it would not be usable). Although it was used to produce Ag-nanoparticle, *G. pentaphyllum* is only rarely reported for its antimicrobial properties [24]. Still, antimicrobial activity has been reported against *Salmonella*, *Vibrio*, *Escherichia*, and *Shigella*; however, the concentration of extract used in the study was much higher than those used here [25]. This again fits well with our observations and measurements. However, *G. pentaphyllum* is known for its antioxidant, antitumoral, hepatoprotective, immunomodulatory, anti-diabetic and anti-fatigue properties [26]. The consumption and depletion of energy sources as well as the production and accumulation of metabolic products including specific polysaccharides are believed to be responsible for anti-diabetic and anti-fatigue properties [26]. Indeed, one might suspect that the extract provides polysaccharides or micronutrients (metals such as iron or zinc) promoting microbial growth. The growth promotion observed stimulates the idea that the *G. pentaphyllum* extract can be used as prebiotics (i.e., to promote growth of beneficial microorganisms) [27]. Indeed, when repeating the experiment with commonly used probiotic strains the growth promotion on those was quite prominent on *Bacillus subtilis* and still rather visible with *Saccharomyces cerevisiae*. For both *B. subtilis* and *S. cerevisiae*, changes in growth parameters were observed. On the contrary of extracts showing an antimicrobial effect characterized by an increase in lag phase, for both samples the increase in lag phase was minimal (1.4 h for *B. subtilis* and 0.3 h for *S. cerevisiae*). In addition, in both cases, there was an increase in the amount of heat released, suggesting a higher growth. For *B. subtilis* the amount of heat was increased by up to 200%, as for *S. cerevisiae* the increase was only by 17%. Finally, the increase in growth rate was clearly visible for *B. subtilis* with an increase in growth rate up to 83%. For *S. cerevisiae* a minor decrease of 12% was observed. Dose dependency was not assessed as only two concentrations were used. However, it appears that the lowest concentration used (7.8 mg·mL^−1^) was more efficient in stimulating growth (Supplementary Table S2). Still, to establish a true value of this extract as prebiotic, the selective stimulation of growth and/or activity(ies) on a limited number of microbial genera according to the definition of prebiotic should be further investigated; however the antimicrobial activity against *Salmonella*, *Vibrio*, *Escherichia*, and *Shigella* observed in the literature [25] combined with the growth-promoting effect measured here warrants such further investigations as selective antimicrobial and growth-promoting effect can be shown. Such future studies should however use the same extract for a clear demonstration. In addition, the nature of growth-promoting compounds produced should be investigated as carbohydrate-based (e.g., inulin, fructooligosaccharides (FOS), galactooligosaccharides (GOS), and xylooligosaccharides (XOS)) as well as some non-carbohydrate compounds such as polyphenols, minerals, and polyunsaturated fatty acids have been proposed as prebiotic candidates [28,29].

Methodology-wise isothermal microcalorimetry provides a quantitative assessment of the antimicrobial or growth-promoting activity of the extract tested. On the contrary to optical methods (spectrophometry, fluorometry or even microscopy), darkness or cloudiness of the extract is not limiting the measurement ability of microcalorimeters. Isothermal microcalorimetry provides also data comparable to conventional testing using culture on plates (disk-diffusion or well-diffusion methods). Thus we believe that isothermal microcalorimetry could become valuable in the study of hairy root and/or callus cultures.

## 4. Materials and Methods

### 4.1. Hairy Root Culture and Hairy Root Extracts

Hairy roots were originally obtained using decontaminated seeds exposed to Rhizobium rhizogenes (formerly Agrobacterium). The original seeds were obtained from Emmentaler Ginseng (Affoltern, Switzerland) for *Panax. ginseng* Mey., Mediseeds (Contey, Switzerland) for *Leontopodium alpinum* Cass. and Strictly Medicinal LLC (Williams, OR, USA) for *Gynostemma pentaphyllum* Thunb.

**Figure 2:**
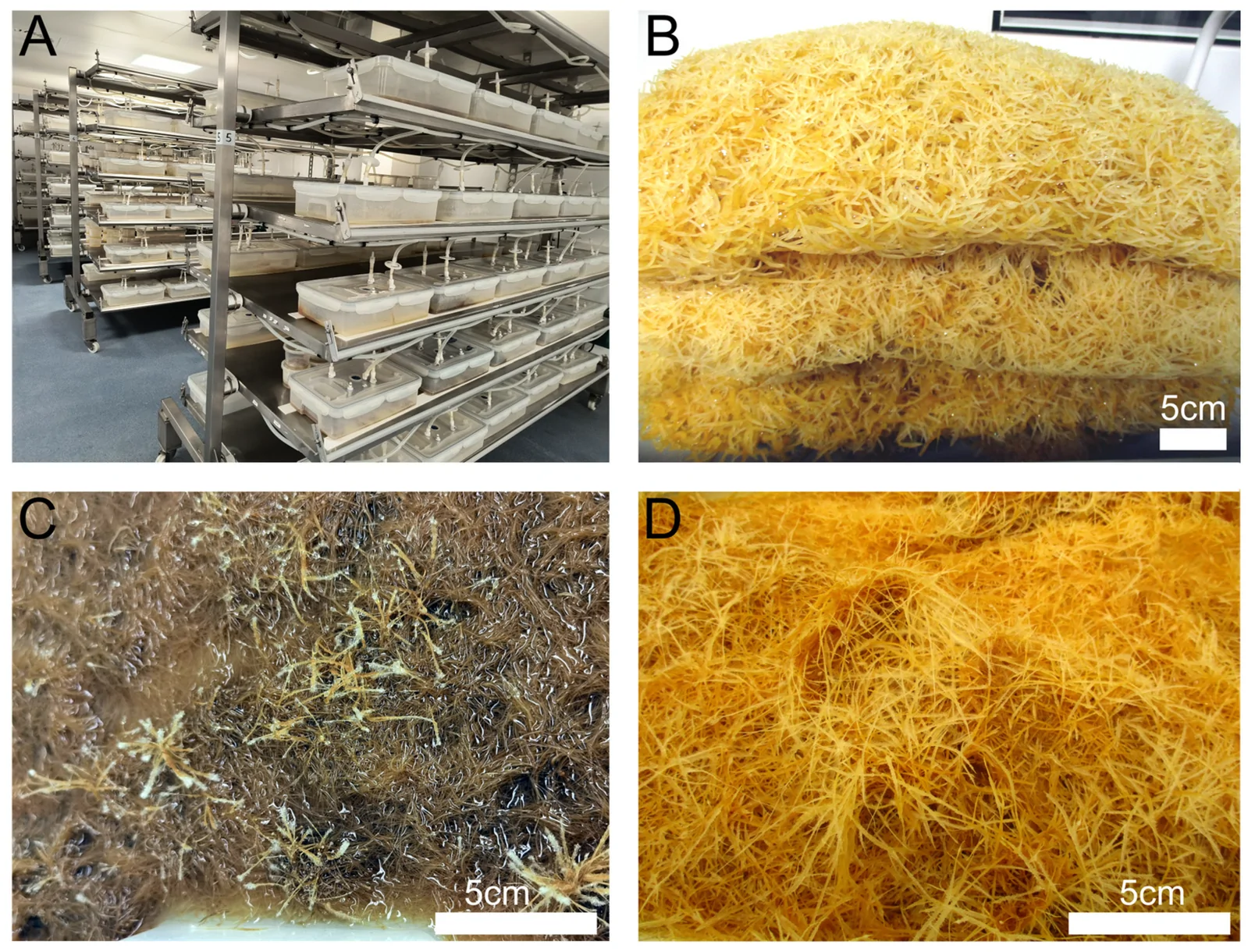
General view of the production area and resulting products. (**A**) production area showing incubators and trays of inoculated medium in the temperature and humidity-controlled room. (**B**) Biomass harvested from *P. ginseng*. (**C**) Biomass harvested from *L. alpinum*. (**D**) Biomass harvested from *G. pentaphyllum*.

Inocula of the three plant species (*P. ginseng*, *L. alpinum*, and *G. pentaphyllum*) are cultivated in a MS-based medium (KNO_3_ 1.900 mg·L^−1^, NH_4_NO_3_ 1.650 mg·L^−1^, CaCl_2_*2H_2_O 440 mg·L^−1^, MgSO_4_*7H_2_O 370 mg·L^−1^, KH_2_PO_4_ 170 mg·L^−1^, Na_2_.EDTA*2H_2_O 37.2 mg·L^−1^, FeSO_4_*7H_2_O 27.8 mg·L^−1^, MnSO_4_*4H_2_O 22.3 mg·L^−1^, ZnSO_4_*7H_2_O 8.6 mg·L^−1^, H_3_BO_3_ 6.2 mg·L^−1^, KI 0.83 mg·L^−1^, CoCl_2_*6H_2_O 0.025 mg·L^−1^, Na_2_MoO_4_*2H_2_O 0.25 mg·L^−1^, CuSO_4_*5H_2_O 0.025 mg·L^−1^—[30]) added with NovaPhyRT^®^ NPRT3 proprietary supplement of DXC-RT (DXC-RT, Aesch, Switzerland). Cultures were performed in dedicated bioreactors (Figure 1A—(DXC-RT, Aesch, Switzerland)) previously cleaned with bleach and sterilized by autoclaving (121 °C for 15 min). All handling was performed under a laminar flow cabinet. All the material used is sterilized by autoclaving before the transfer of the inoculum and cleaning with bleach thereafter. Finally, the hairy root cultures are grown during 4 to 6 weeks in the dark (except for culture maintenance operations–ca 16 h per week) at a temperature maintained between 22 °C and 24 °C and a relative humidity comprised between 40 and 50% within a controlled room. Finally, hairy roots are harvested as shown in Figure 2.

### 4.2. Hairy Root Biomass Methanolic Extraction

Once collected hairy roots were air dried at 35 °C and ground in a mortar. The extracts used for this study were prepared using 2 g of obtained dry powder. The powder was placed in a 50 mL falcon tube and added with 20 mL of High Performance Liquid Chromatography (HPLC)-grade methanol. The samples were first sonicated for 30 min at 35 °C. After sonicating, samples were centrifuged for 10 min at 2500 rpm speed. The supernatant was transferred in a new tube and evaporated on a heating plate (around 8 h at 65 °C) until all samples reach dryness. This procedure led to the recovery of 142 mg of dried extract for *P. ginseng*, 615 mg for *G. pentaphyllum* and 607 mg for *L. alpinum*. Note that the elevated temperature used during drying has been shown to be compatible with the extraction of ginsenoside and various compounds from *L. alpinum* [31,32,33]—in particular linolic and linoleic acids providing antimicrobial activity. Still, other molecules providing anti-oxidant effects for example might not be preserved by such treatment.

### 4.3. Microbial Culture

*Staphylococcus epidermidis* (American Type Culture Collection—ATCC 49461) was used in this study as this strain is commonly used in antimicrobial testing and quality control research. Stocks of the bacteria in Luria broth (LB) medium added with 20% (*v*/*v*) glycerol were stored at −80 °C. Before each experiment, an aliquot was unfrozen and used as inoculum for an overnight culture performed in LB medium at 37 °C. The optical density (OD_600_) of the culture was measured and appropriate dilutions were made to reach the target concentration of ca 10^5^ colony forming unit per mL (CFU·mL^−1^) for further use.

### 4.4. Isothermal Microcalorimetry

Sterile 4 mL microcalorimetric ampoules were filled with 3 mL of freshly diluted overnight culture (see above) and added with serial 2-fold dilution of the hairy root extract resuspended in ethanol:PBS mixture (1:1). The concentrations of extract used ranged from 31 mg·L^−1^ to 0.9 mg·L^−1^ depending on the extract (see Figure 1). Inoculated ampoules without hairy root extracts were prepared as the control group. The prepared ampoules were crimp sealed and introduced in a TAM48 (Waters/TA, New Castle, DE, USA) microcalorimeter previously set and equilibrated at least for two days at 37 °C. After the recommended two-step thermal equilibration procedure, the heatflow was measured until the signal returned to baseline. Controls were prepared with the same volumes of ethanol:PBS mixture without the hairy root extracts. A maximum concentration of ethanol of 2.5% (*v*/*v*) was used and was previously shown not to affect *S. epidermidis*. All measurements were performed in triplicates.

### 4.5. Data Analysis

Calorimetric raw data (heatflow or thermal power) were integrated to obtain the heat-over-time curve. The heat-over-time was fitted with the Gompertz growth model as described previously [34]. The growth rate (μ), lag phase (λ) and total heat produced (Q) were extracted. After normality of the data was tested using the Shapiro–Wilk test, normally distributed parameters were compared using *t*-test or ANOVA when appropriate. Non-parametric test (Kruskal–Wallis and Mann–Whitney U-test) were used for the other parameters. All calculations were performed with R (3.6.3) and the grofit package [35,36]. The MIC was determined as the first concentration that fully inhibited growth in the calorimeter (i.e., heatflow remained at baseline level for the duration of the experiment).

### 4.6. Kirby–Bauer Disk Diffusion Assay

**Figure 3:**
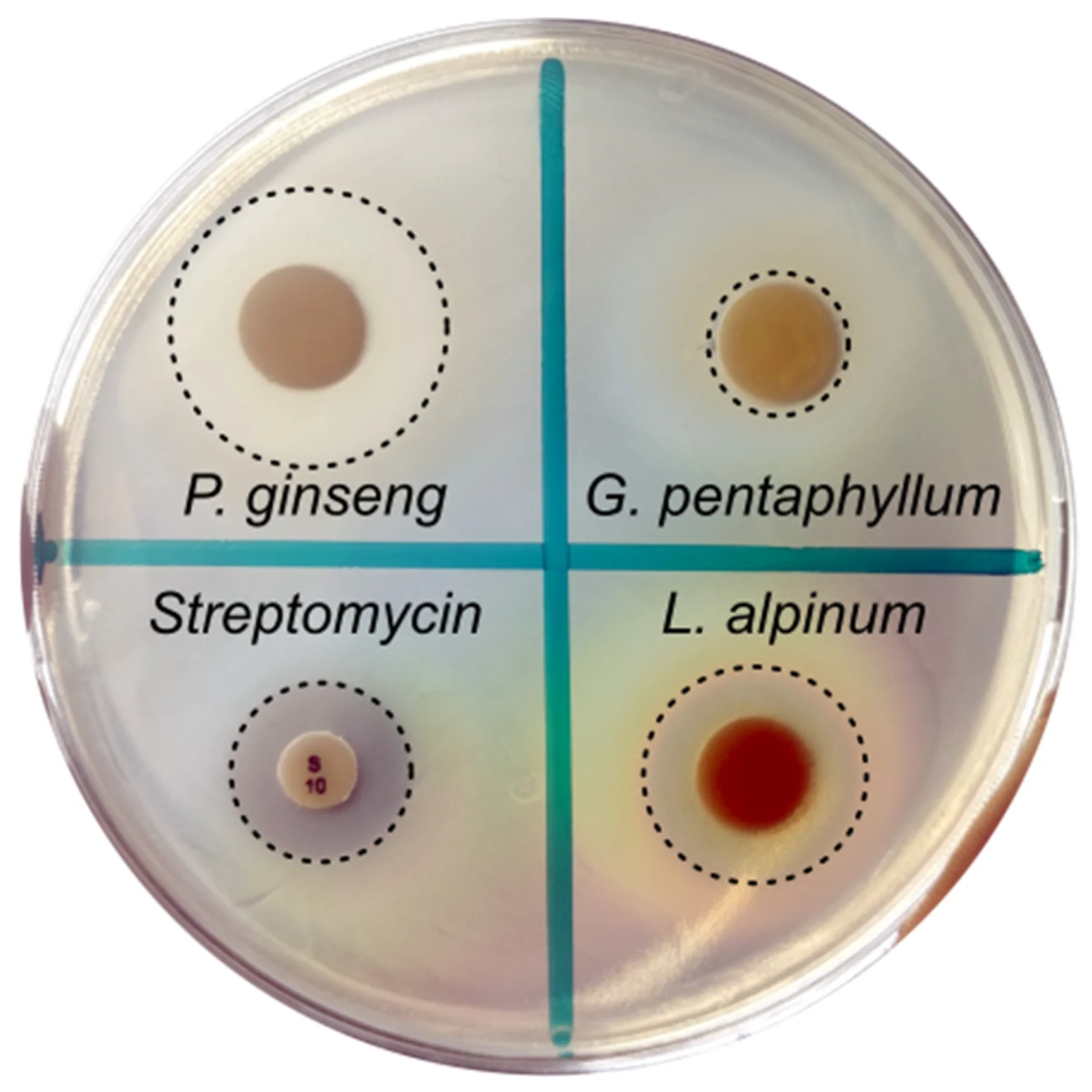
An example of a plate used in the Kirby–Bauer assay showing area of inhibition (dashed line added for visibility due to the low contrast) for *P. ginseng*, *G. pentaphyllum* and *L. alpinum*; streptomycin added as positive control.

Hairy root biomass methanolic deposited onto sterile 10 mm disks. The same extract was used as for calorimetric measurements leading to the following extract amount per disk: 21 μg_dw_/disk for *P. ginseng*, 93 μg_dw_/disk for *L. alpinum* and 91 μg_dw_/disk for *G. pentaphyllum*. The disks were placed for 10 min in the biosafety cabinet to allow for ethanol evaporation. At this point the disks were placed on previously inoculated LB plates that were further incubated for 24 h at 35 ± 2 °C. Positive controls were made using a commercial antimicrobial testing disk containing 10 μg_dw_/disk of streptomycin. Sterile unloaded disks were used as negative controls. The inhibition zone if any was measured and recorded (see example in Figure 3). No inhibition zone could be seen for the negative controls.

## 5. Conclusions

Industrial callus culture and hairy root production are a valuable alternative to the use of field-grown plants and allows the access to new botanical resources. Using isothermal microcalorimetry as a platform antimicrobial effect of *P. ginseng* and *L. alpinum* as well as prebiotic effects of *G. pentaphyllum*, hairy root extracts were accurately measured using changes in growth rate, lag phase and metabolic heat of the treated microbes. Effects similar to those described in many pharmacopeias were observed. Still, a direct experimental comparison would be needed to ascertain similar effects of the hairy root extract and extract from their field-grown counterparts. This emphasizes that isothermal microcalorimetry provides a valuable tool as it can rapidly, quantitatively and accurately record changes in indicating antimicrobial or prebiotic effects even in dark or cloudy samples as those used in this study and can be used in research but also for quality control purposes.

## Figures and Tables

**Table 1:** Summary of the disk diffusion assay (Kirby–Bauer assay) for the different extract and streptomycin as a reference antimicrobial (i.e., positive control).

	*P. ginseng*	*L. alpinum*	*G. pentaphyllum*	Streptomycin
Mass per disk	21 μg_dw_	93 μg_dw_	93 μg_dw_	10 μg_dw_
Inhibition Ø	21 ± 1 mm	21 ± 3 mm	13 ± 1 mm	17 ± 1 mm
n (replicates)	4	4	4	5

“Ø” represents diameter.

**Table 2:** Amount of ginsenoside present in root and hairy roots expressed in mg of ginsenoside per gram ginseng (dry weight). The table provides a comparison with hairy roots produced in the DXC-RT pilot plant. Note that care should be taken when comparing those results as different extraction methods were used by the different authors cited in the table. (NA not available; ND not determined).

Ginsenoside	Li et al. ^1^ [21]	Chen et al. ^2^ [20]	Shi et al. ^3^ [22]	DXC-RT *
Rb1	15.9	19.9	16.2	19.5
Rb2	3.8	8.2	12.4	18.0
Rb3	0.6	1.7	1.9	ND
Rg1	6.5	15.6	4.4	16.8
Re	6.2	11.7	25.9	27.4
Rf	NA	4.0	NA	2.8

^1^ Values for field-grown plant root hairs; ^2^ batch with the highest total ginsenoside (field-grown 5-year-old root); ^3^ batch with the highest total ginsenoside (field-grown plants); and * independent analysis performed in an outside laboratory.

## Data Availability

The data that support the findings of this study are available from the corresponding author, [OB], upon reasonable request.

## Supplementary Materials

The following supporting information can be downloaded at: https://www.mdpi.com/article/10.3390/antibiotics15090905/s1. Supplementary Table S1. Growth parameters measured using calorimetric data during the growth of *S. epidermidis* in LB medium added with increasing concentrations of the various extracts (*P. ginseng*, *L. alpinum*, *G. pentaphyllum*) as well as references antimicrobial (streptomycin) and sterile controls. NA not available. Values in parenthesis indicate that due to the very little growth data are to be taken with care due to the high standard deviations; Supplementary Table S2. Growth parameters measured using calorimetric data during the growth of commonly used probiotics in LB medium (for *B. subtilis*) and YPD medium (for *S. cerevisiae*) added with increasing concentrations of *G. pentaphyllum* extract. Sterile controls (Blanks) were performed using the same media and data were pooled. NA not available.
