# Supplementary material for: Hairy Roots Cultures from the Slow-Growing and Protected Panax ginseng, Leontopodium alpinum and Gynostemma pentaphyllum as an Alternative Source of Antimicrobials
Source: Antibiotics (Basel). 2026 Sep 14;15(9):905. doi: 10.3390/antibiotics15090905 (PMC13603935; doi:10.3390/antibiotics15090905)
Supplement: Supplementary file 1 [file antibiotics-15-00905-s001.zip › antibiotics-4417568-supplementary.pdf]

antibiotics

MDPI

Article

Hairy roots cultures from the slow-growing and protected *Panax ginseng, Leontopodium alpinum and Gynostemma Pentaphyllum* as an alternative source of antimicrobials

Olivier Braissant[1],*Virginie Flury[2], Muriel Jobin[2], Sophie Hu [2,*]

[1] Dept. Biomedical Engineering, Univ. of Basel, Hegenheimermattweg 167 B/C, CH-4123 Allschwil, Switzerland; olivier.braissant@unibas.ch
[2] DXC RT AG, Gewerbestrasse 7a, 4147 Aesch, Switzerland
* Correspondence: olivier.braissant@unibas.ch

Supplementary material

Supplementary table S1. Growth parameters measured using calorimetric data during the growth of *S. epidermidis* in LB medium added with increasing concentrations of the various extracts (*P. ginseng, L. alpinum, G. pentaphyllum*) as well as references antimicrobial (streptomycin) and sterile controls. NA not available. Values in parenthesis indicate that due to the very little growth data are to be taken with care due to the high standard deviations.

Sample	Concentration†	Growth rate [µ]	Lag phase [h]	Heat [J]	n
Controls*	0	0.10 ± 0.01	2.3 ± 0.3	6.7 ± 0.7	6
P. ginseng	0.9	0.08 ± 0.01	0.3 ± 10.0	7.7 ± 1.7	3
	1.8	0.06 ± 0.03	5.7 ± 6.1	7.3 ± 0.3	3
	3.6	0.00 ± 0.00	39.9 ± 9.8	0.3 ± 0.1	3
	7.1	0.00 ± 0.00	NA	0.3 ± 0.1	3
L. alpinum	3.8	0.13 ± 0.03	5.6 ± 10.0	13.2 ± 1.3	3
	7.6	0.09 ± 0.09	32.5 ± 6.1	7.8 ± 7.2	3
	15.2	0.01 ± 0.01	(11.4 ± 13.25)	2.6 ± 1.8	3
	30.3	0.00 ± 0.00	NA	0.3 ± 0.0	3
G. penthaphyllum	3.9	0.21 ± 0.06	7.2 ± 2.9	9.2 ± 1.2	3
	7.8	0.24 ± 0.01	10.9 ± 0.5	11.6 ± 0.3	3
	15.5	0.21 ± 0.06	10.5 ± 3.2	11.3 ± 0.1	3
	31.0	0.18 ± 0.14	17.3 ± 9.8	7.8 ± 2.1	3
Streptomycin	2	0.10 ± 0.01	0.2 ± 0.4	6.7 ± 0.6	3
	4	0.08 ± 0.01	0.1 ± 0.9	7.2 ± 0.3	3
	8	0.05 ± 0.01	16.9 ± 1.3	5.6 ± 0.0	3
	16	0.05 ± 0.02	65.5 ± 4.3	1.3 ± 0.3	3
	32	0.00 ± 0.00	NA	0.1 ± 0.0	3

 https://doi.org/10.3390/antibiotics15090905

Blanks (sterile)	0	0.00 ± 0.00	NA	0.50 ± 0.02	6

* Pooled controls

† Concentrations are expressed in mg/L except for streptomycin in ug/L

Supplementary table S2. Growth parameters measured using calorimetric data during the growth of commonly used probiotics in LB medium (for *B. subtilis*) and YPD medium (for *S. cerevisiae*) added with increasing concentrations of *G. pentaphyllum* extract. Sterile controls (Blanks) were performed using the same media and data were pooled. NA not available. .

Sample	Concentration†	Growth rate [µ]	Lag phase [h]	Heat [J]	n
B. subtilis	0	0.59 ± 0.04	5.3 ± 0.3	5.40 ± 1.3	3
	7.8	1.04 ± 0.03	6.7 ± 0.2	16.3 ± 0.6	3
	15.5	0.77 ± 0.01	5.52 ± 0.12	11.6 ± 0.15	3
S. cerevisiae	0	3.23 ± 0.03	4.1 ± 0.0	31.8 ± 0.3	3
	7.8	2.82 ± 0.09	4.4 ± 0.1	37.3 ± 0.2	3
	15.5	2.92 ± 0.01	4.4 ± 0.5	33.8 ± 1.2	3
Blanks (sterile)	0	0.01 ± 0.01	NA	0.3 ± 0.1	6

† Concentrations are expressed in mg/L

Disclaimer/Publisher's Note: The statements, opinions and data contained in all publications are solely those of the individual author(s) and contributor(s) and not of MDPI and/or the editor(s). MDPI and/or the editor(s) disclaim responsibility for any injury to people or property resulting from any ideas, methods, instructions or products referred to in the content.

https://doi.org/10.3390/antibiotics15090905
